# A fatal case of spinal tuberculosis mistaken for metastatic lung cancer: recalling ancient Pott's disease

**DOI:** 10.1186/1476-0711-8-32

**Published:** 2009-11-20

**Authors:** Felix C Ringshausen, Andrea Tannapfel, Volkmar Nicolas, Andreas Weber, Hans-Werner Duchna, Gerhard Schultze-Werninghaus, Gernot Rohde

**Affiliations:** 1 Department of Medicine III, Pneumology, Allergology and Sleep Medicine, University Hospital Bergmannsheil, Ruhr-University Bochum, Germany; 2 Department of Medicine, Spital Bülach, Bülach, Switzerland; 3 Institute of Pathology, Ruhr-University Bochum, Germany; 4 Institute of Diagnostic Radiology, Interventional Radiology and Nuclear Medicine, University Hospital Bergmannsheil, Ruhr-University Bochum, Germany

## Abstract

**Background:**

Tuberculous spondylitis (Pott's disease) is an ancient human disease. Because it is rare in high-income, tuberculosis (TB) low incidence countries, misdiagnoses occur as sufficient clinical experience is lacking.

**Case presentation:**

We describe a fatal case of a patient with spinal TB, who was mistakenly irradiated for suspected metastatic lung cancer of the spine in the presence of a solitary pulmonary nodule of the left upper lobe. Subsequently, the patient progressed to central nervous system TB, and finally, disseminated TB before the accurate diagnosis was established. Isolation and antimycobacterial chemotherapy were initiated after an in-hospital course of approximately three months including numerous health care related contacts and procedures.

**Conclusion:**

The rapid diagnosis of spinal TB demands a high index of suspicion and expertise regarding the appropriate diagnostic procedures. Due to the devastating consequences of a missed diagnosis, Mycobacterium tuberculosis should be considered early in every case of spondylitis, intraspinal or paravertebral abscess. The presence of certain alarm signals like a prolonged history of progressive back pain, constitutional symptoms or pulmonary nodules on a chest radiograph, particularly in the upper lobes, may guide the clinical suspicion.

## Background

In 2007, one-fifth of 5,020 tuberculosis (TB) cases reported to the responsible German authority (Robert Koch Institute) were extrapulmonary, mainly lymphatic (9.1%) disease manifestations, but only 0.8% of all adult TB cases were spinal TB [1]. Even though spinal TB is scarce in countries with a low incidence of TB, it is an ancient human disease. While the characteristic clinical features of tuberculous spondylitis were first described in the medical literature by Sir Percivall Pott in 1779 (Pott's disease) [2], spinal TB has been identified in Egyptian mummies dating back to 3000 B. C. by typical skeletal lesions and consecutive DNA analysis [3]. A delay in diagnosis and timely initiation of treatment of aggressive TB manifestations like central nervous system (CNS) TB or vertebral TB may cause severe and irreversible neurologic sequelae including paraplegia, even if antimycobacterial chemotherapy is available [4,5]. Moreover, as evidence of either previous or current pulmonary TB is found in approximately half the reported patients, a delay in diagnosis may lead to further significant exposure of contacts, particularly endangering health care workers and thus highlighting TB's potential as a nosocomial and occupational disease [6].

We report a fatal case of a patient with Pott's disease, who was misdiagnosed and irradiated for metastatic lung cancer of the spine. Subsequently, the patient progressed to CNS TB, and finally, disseminated TB before the accurate diagnosis was established after an in-hospital course of approximately three months including numerous health care related contacts and procedures [7].

## Case presentation

**Figure 1:**
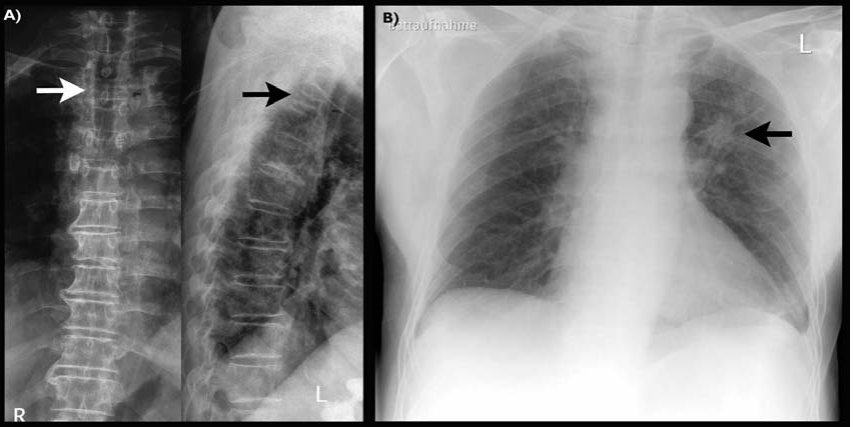
**Conventional radiographs of the thoracic spine and the chest on initial presentation**. (A) Compression fracture of the third thoracic vertebra (arrows). (B) Solitary pulmonary nodule of 2.5 cm in diameter of the left upper lobe (arrow), in posterior-anterior and lateral projection, respectively.

**Figure 2:**
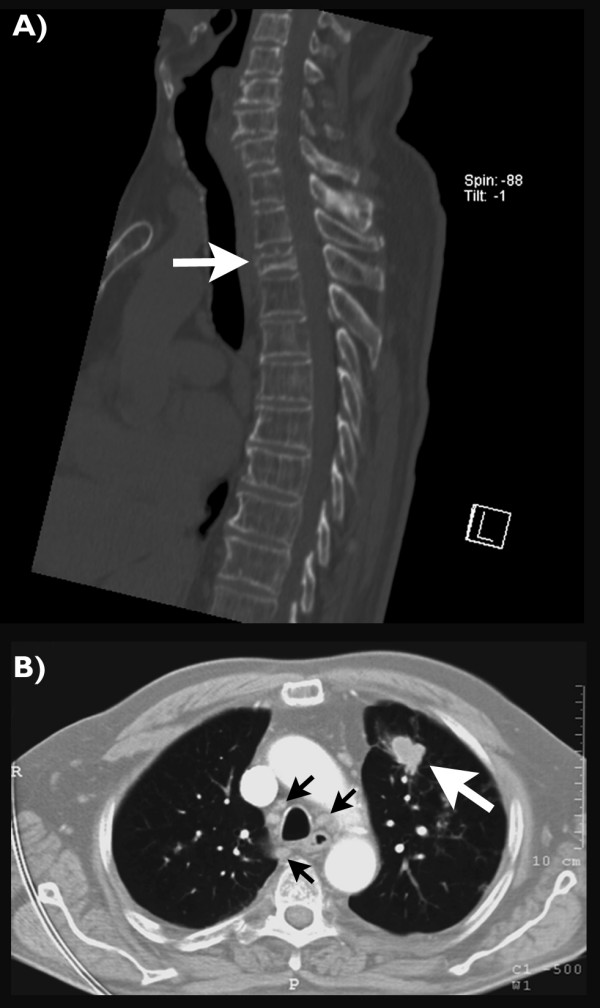
**CT scans of the spine and the chest**. The sagittal reconstruction of the native CT scan of the spine (A) confirms a compression fracture of the third thoracic vertebra with lytic destructions of posterior margins of both adjacent vertebral bodies (arrow). The axial reconstruction of the contrast enhanced CT scan of the chest (B) suggests a tumorous lesion of the ventral left upper lobe (large arrow) with accompanying mediastinal lymphadenopathy (small arrows).

A 67-year old Caucasian male of German descent presented to the emergency department of an external hospital at December 8^th ^2006 due to severe upper back pain. His past medical history was unremarkable. He had no history of previous TB or TB exposure. His complaints had emerged over the past six month and were accompanied by slight night sweats and a moderate weight loss. Physical examination and routine laboratory studies on admission including hemoglobin, white blood count and C-reactive protein (CRP) were within normal range. Conventional radiographs of the chest and the thoracic spine revealed a compression fracture of the third thoracic vertebra (Th3) and a solitary pulmonary nodule of the left upper lobe (Figure 1). On the basis of this coincidence, a pathological vertebral fracture secondary to metastatic lung cancer was suspected. The further work-up included a computed tomography (CT) scan of the chest and of the spine, which confirmed the thoracic vertebral compression fracture. Moreover, it revealed lytic destructions of the posterior margins of both adjacent vertebral bodies and showed an intraspinal soft tissue mass expanding between Th2 and Th5. Additionally, a tumorous lesion of the ventral left upper lobe was observed. Apparent mediastinal lymphadenopathy was interpreted as metastatic disease of the mediastinal lymph nodes (Figure 2). Bone scintigraphy was not suggestive of malignancy and detected only minor degenerative alterations of the upper spine. Both repeated transbronchial biopsies, which had been obtained by flexible bronchoscopy, and repeated CT-guided transthoracic needle biopsies of the pulmonary nodule failed to establish a specific histological diagnosis, and were read as "necrosis compatible with necrotic tumor tissue".

Given the progressive back pain and supposed spinal instability, the patient was referred to another external hospital, where a palliative irradiation of the upper thoracic spine was initiated and a cumulative dose of 36 Gy was applied over three consecutive weeks. However, the patient's condition deteriorated and he soon developed paresthesia and paralysis of both lower limbs. At this point, magnetic resonance imaging (MRI) of the spine revealed an extensive intraspinal abscess, which resulted in compression of the upper thoracic spinal cord. Hence, the patient was immediately referred to the neurotraumatological service of our university hospital for surgical treatment.

**Figure 3:**
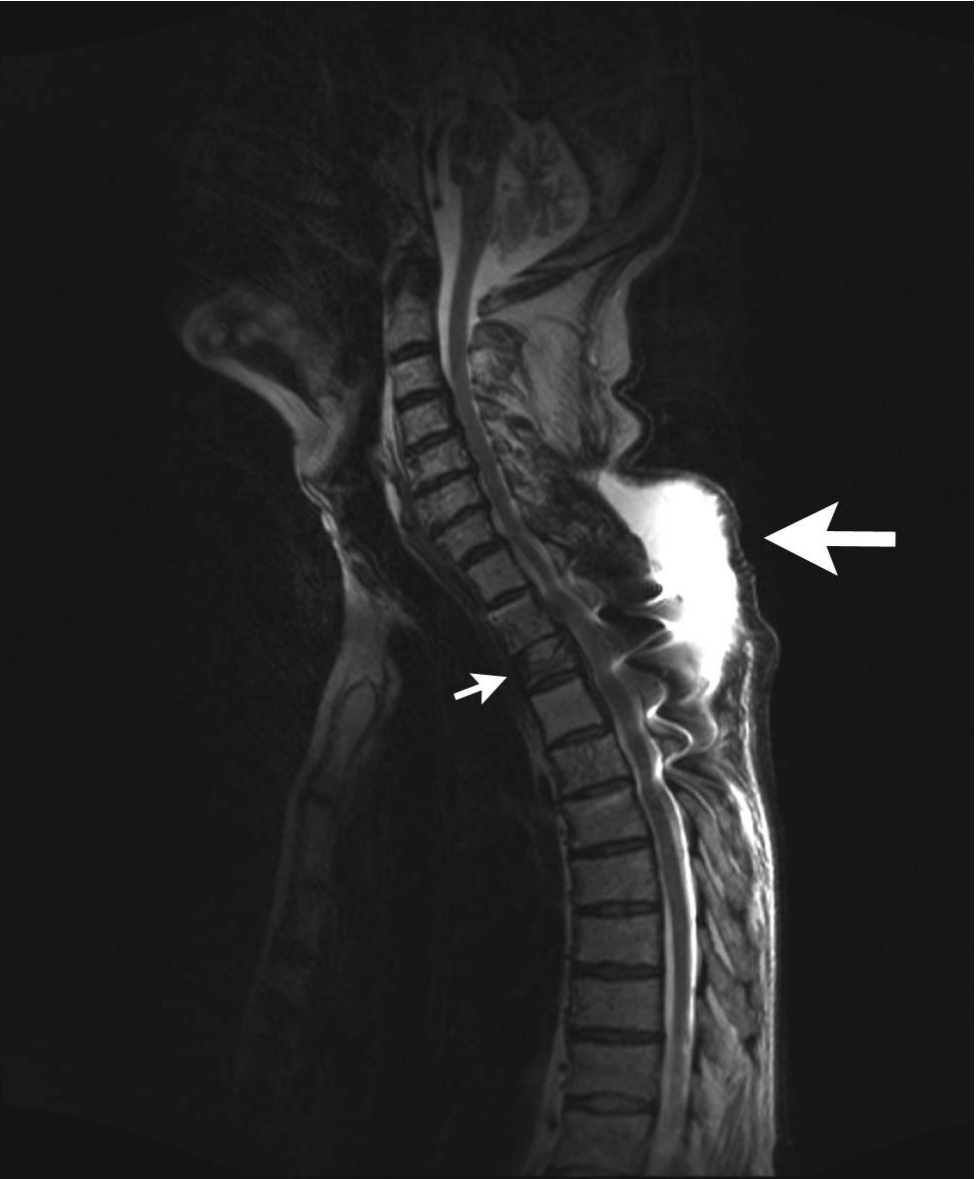
**MRI of the thoracic spine (T2-weighted, sagittal reconstruction)**. The dorsal fluid collection suggests a paravertebral abscess (large arrow) just above the fractured and operated third thoracic vertebra (small arrow).

**Figure 4:**
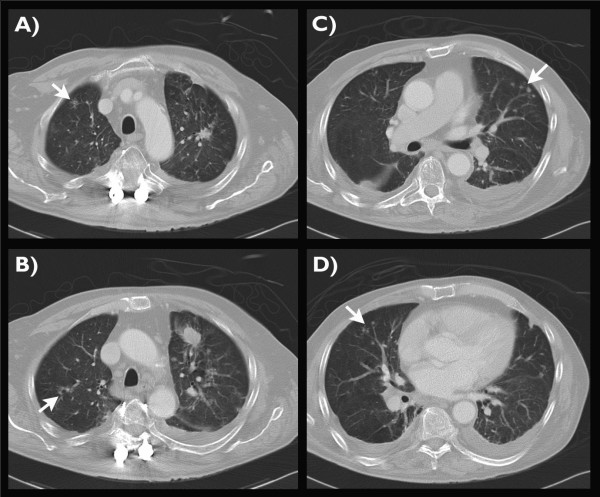
**Follow-up contrast enhanced CT scan of the chest**. The axial CT reconstructions in craniocaudal sequence (A-D) show exudative pulmonary tuberculosis with multiple novel opacities and tree-in-bud sign (arrows). Secondary findings: post-specific scarring (A), tuberculoma (B) of the left upper lobe, and small bilateral pleural effusions (B-D).

Upon arrival at our institution on January 10^th ^2007 the patient presented with incomplete paraplegia at the level of Th3. Promptly, spinal decompression surgery including laminectomy of Th2 to Th4, revision of vertebra Th3, and thoracic spondylodesis from Th1 to Th6 was performed. The histology of the removed intraspinal soft tissue mass was read as an unspecific chronic inflammatory and granulating process. No malignancy or specificity was evident. Routine cerebrospinal fluid (CSF) cultures were sterile. No further CSF analyses were performed. Soon, after an initial post-operative improvement, the patient deteriorated again and developed a persistent dorsal swelling, reddening, and fluid collection that was suggestive of a paravertebral abscess (Figure 3). Thus, surgical revision was repeated. Again, routine CSF cultures grew no microorganisms and no further CSF analyses were prompted. Nevertheless, temperatures >38.0°C persisted despite the use of various antimicrobial regimes. Repeated routine blood cultures remained sterile and infectious endocarditis was ruled out by transesophageal echocardiography. A CT scan of the brain, which was performed due to persistent headaches, showed no abnormalities, while a follow-up CT scan of the chest showed multiple novel opacities with tree-in-bud sign (Figure 4).

When the patient was finally referred to the pulmonary department for further evaluation of the initially suspected metastatic lung cancer on February 28^th ^2007, he had developed a new brachiofacially accentuated, right-sided hemiparalysis and aphasia. Now, MRI of the brain revealed multiple fresh ischemic bihemispheric and pontine lesions. Again, infectious endocarditis was ruled out.

The patient was febrile with a temperature of 39.3°C despite an antimicrobial regime consisting of Imipeneme/Cilastatin, Vancomycin, Ciprofloxacin, and Metronidazole, which had been administered for the past two weeks. At this time, the major findings on physical examination included cachexia, signs of respiratory distress (a respiratory rate of 25/min, productive cough, but insufficiently coughing up), moderate fluid overload (bilateral basal pulmonary rales, peripheral edema), and a dorsal paravertebral reddish and fluctuating swelling at the site of prior surgery, but no meningeal signs. Furthermore, we observed oral candidiasis and a unilateral segmental herpes zoster rash at the lower back that both indicated relevant immunosuppression. Now, routine laboratory studies showed markedly increased inflammatory activity (CRP 22 mg/dL), but a normal white blood count of 4,900/μL. HIV serology was negative. Remarkably, the interferon-γ release assay QuantiFERON^®^-TB Gold In-Tube was negative (IFN 0.189 IU/mL).

However, complicated and disseminated TB with bone, soft tissue, CNS, and pulmonary involvement was suspected by thoroughly reviewing the patient's previous history and medical course. We immediately prompted retrospective polymerase chain reaction (PCR) analysis of the surgically resected tissue regarding the presence of Mycobacterium-tuberculosis-(MTB)-complex DNA and initiated microbiological sampling for the confirmation of the suspected diagnosis. Aspirates of the dorsal fluid collection suggested an abscess (purulent appearance, low glucose concentration of 0.3 mmol/L), but no CSF fistula (low β-trace-protein of 3.8 mg/L). No microorganisms were detected on gram stain or acid-fast bacilli (AFB) smear. CSF was obtained by lumbar puncture and showed signs of purulent meningitis (800/μL predominantly polymorphonuclear cells, low glucose concentration of 0.8 mmol/L, elevated protein level of 1.5 g/L). CSF microscopy (gram stain and AFB smear), routine cultures, and PCR for MTB-complex DNA were negative. As the patient was suffering from severe respiratory distress, bronchoscopy was considered potentially harmful and therefore abandoned. Instead, repeated morning fasting gastric acid aspirates, which may be considered as swallowed respiratory secretions according to the German authorities [1], were obtained for TB cultures in order to evaluate for the dissemination of viable mycobacteria via the respiratory route. AFB smears of repeated gastric acid aspirates were negative. Subsequently, both PCR results of the resected intraspinal tumor tissue and the aspirate of the paravertebral abscess indicated the presence of MTB-complex DNA. After two weeks TB cultures grew MTB from both the abscess and gastric acid aspirates and after six weeks from CSF, too. The MTB isolate was fully susceptible to all first line antimycobacterial drugs. All samples had been negative on AFB smears with both auramine and Ziehl-Neelsen staining (Table 1).

**Table 1:** Summary of microbiological results

	Method		
	
Specimen	AFB smear	PCR*	**TB Culture**^†^
Spinal tumor mass	NA	positive	NA
Paravertebral abscess	negative	positive	positive
Morning fasting gastric acid^‡^	negative	NA	positive
Cerebrospinal fluid	negative	negative	positive

*PCR amplified gene fragments specific for Mycobacterium-tuberculosis-complex mycobacteria (insertion sequence IS6110). ^†^Cultures grew Mycobacterium tuberculosis fully susceptible for all first line antituberculous drugs; ^‡^Morning fasting gastric acid was considered as swallowed respiratory secretions according to the German authorities [1]. AFB = acid-fast bacilli; NA = not assessed; PCR = polymerase chain reaction; TB = tuberculosis

**Figure 5:**
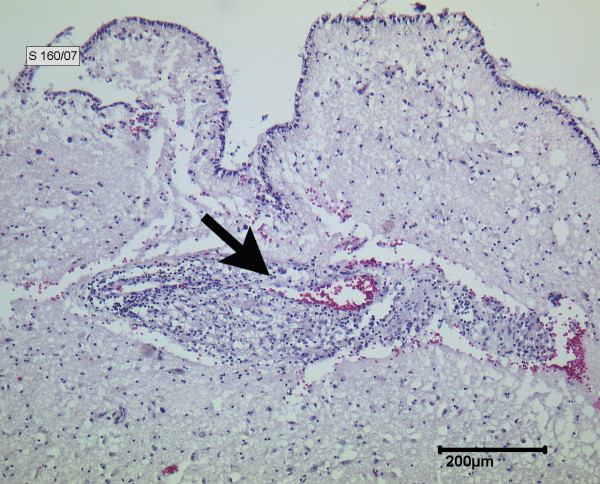
**Tuberculous meningoencephalitis (hematoxylin and eosin stain)**. High-grade active and chronic lymphocytic and granulomatous cerebral vasculitis (arrow).

**Figure 6:**
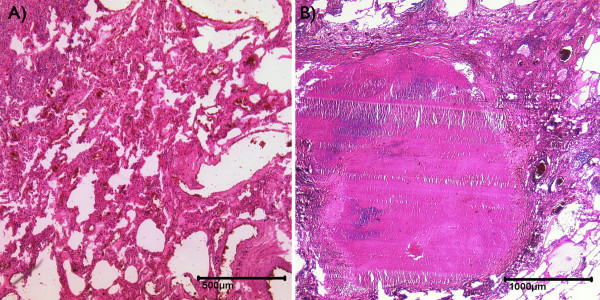
**Pulmonary tuberculosis (hematoxylin and eosin stain)**. Miliary foci with partially active necrotizing epitheloid granulomas (A) besides postspecific scarring with irregular traction emphysema and bronchiolectasia (B).

**Figure 7:**
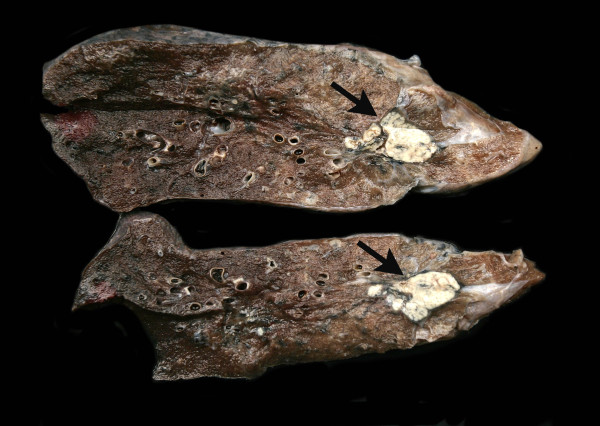
**Macroscopic preparation of the left lung**. Tuberculoma of the left upper lobe (2.5 cm in diameter, arrow).

**Figure 8:**
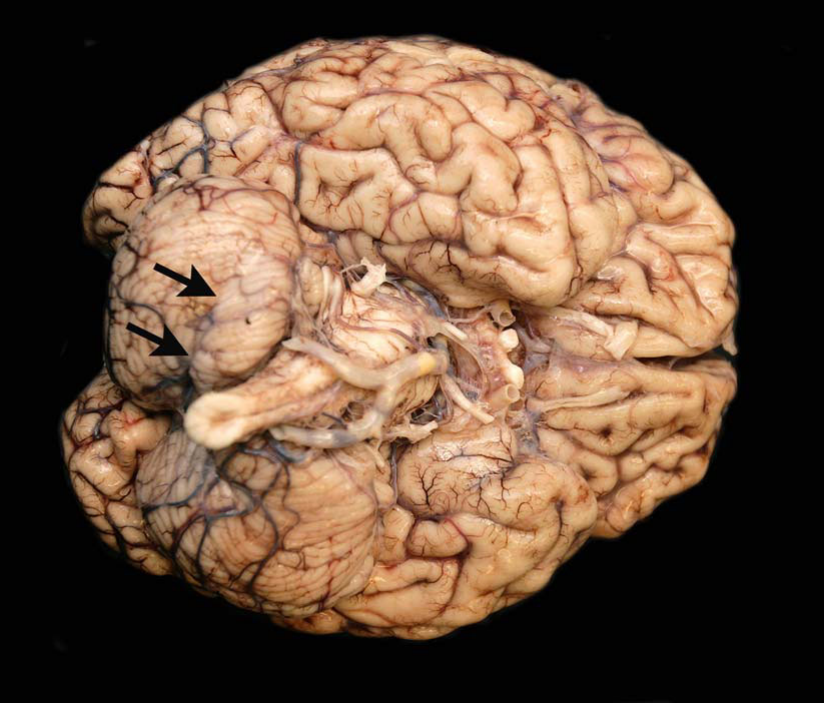
**Macroscopic preparation of the brain demonstrating final cerebral edema**. The arrows indicate a lower cerebral incarceration secondary to increased intracranial pressure, which caused CNS regulation disturbance, and finally, resulted in ventricular fibrillation and cardiac arrest.

On March 7^th ^2007, a total of 57 days after referral to our hospital, and three months after the first presentation to the external hospital, isolation and antimycobacterial treatment with rifampicine, isoniazide, pyrazinamide, and streptomycin were initiated. Due to apparent meningitis, extensive cerebral vasculitis, and suspected arachnoiditis, an adjuvant treatment with oral corticosteroids was administered and tapered after three weeks [8]. Lumbar puncture was repeated one week after treatment initiation and showed clearly improved findings (290/μL mixed mono- and polymorphonuclear cells, negative AFB smear, negative TB cultures after eight weeks). Within the following weeks the patient's general and neurological condition gradually improved. Isolation was terminated and mobilization into a wheel chair was achieved after four weeks of treatment due to intensive physical therapy (See additional file 1: Table S1 - Summary of the patient's diagnostic and therapeutic in-hospital course). Unfortunately, at April 12^th ^2007 the patient died due to a lethal episode of ventricular fibrillation with cardiac arrest. Autopsy confirmed all clinically suspected TB-related diagnoses: spinal TB (Pott's disease) with subsequent tuberculous meningoencephalitis, extensive cerebral vasculitis (Figure 5), spinal arachnoiditis, paravertrebral tuberculous abscess, miliary pulmonary TB (Figure 6), and a tuberculoma of the ventral left upper lobe (Figure 7). Death occurred secondary to CNS dysregulation with elevated intracranial pressure (Figure 8).

## Discussion

There are some important lessons this fatal case teaches. The clinical diagnosis of TB, particularly extrapulmonary TB, requires a certain extent of clinical suspicion and expertise regarding the adequate diagnostic tests. Along with Staphylococcus aureus and a few other bacteria, MTB is still an important cause of spondylitis, intraspinal or paravertebral abscess. In TB low incidence countries spinal TB is mainly a disease of the elderly, resulting from endogenous reactivation of infectious foci that spread during the initial bacteremia. Pott's disease most often affects the lower thoracic and lumbar spine, while disease of the upper thoracic and cervical spine is potentially more disabling [4,9,10]. The most common cause of delay in the diagnosis of Pott's disease is failure to consider the diagnosis. Hence, skeletal TB should always be considered in patients with focal bony abnormalities and a chest radiograph compatible with prior or active TB.

Our patient showed a combination of alarm signals (progressive back pain over several months, presence of a pulmonary nodule of the left upper lobe, night sweats and weight loss), which should have prompted further work-up in order to enforce a definite diagnosis. Back pain is the typical presenting symptom of early Pott's disease, but signs or symptoms of systemic infection are often missing. Constitutional symptoms, fever, and weight loss are unspecific and present in less than 40% of spinal TB cases. Due to the subtle nature of symptoms, diagnostic evaluations are often not initiated until the process is advanced. However, establishing the correct diagnosis is challenging and misdiagnoses may occur in up to 41% of cases [11]. Hence, a significant proportion of patients present with neurological impairment in advanced stages of disease [4,12]. The early changes of spinal TB are particularly difficult to detect by routine radiographs of the spine. CT and MRI scans of the spine are considerably more sensitive and should be obtained whenever an infectious process is suspected [13].

CT-guided percutaneous biopsy of the vertebral body is an effective and safe diagnostic procedure for spinal lesions of unclear origin [14,15]. The consecutive microbiological and histological work-up including PCR may help to establish a rapid diagnosis as PCR results are usually available within one or two working days. In this context, PCR is an extremely useful tool for the guidance of further diagnostic steps and treatment decisions, particularly if the initiation of antimicrobial chemotherapy is crucial. Nevertheless, it should be mentioned that PCR has a limited sensitivity, particularly in AFB smear negative specimens, and that TB culture of clinical specimens remains the gold standard for the confirmation of active TB infection and the assessment of mycobacterial resistance [16].

Combining radical surgery with a standard triple or quadruple antimycobacterial chemotherapy produces the most favourable outcomes and a more rapid neurological recovery [4,10], while a conservative, nonsurgical approach may be warranted in patients without advanced neurological deficits [9,17].

Furthermore, our case emphasizes that IGRAs are of limited value in the context of severely ill patients, where false negative results occur secondary to the host's anergy. IGRAs were developed as immunological diagnostics for the diagnosis of TB infection rather than TB disease, and data on the sensitivity of these assays in critically ill patients and patients with severe immunosuppression and advanced or disseminated active TB is sparse [18].

There were important implications of the delayed diagnosis for the health care workers (HCWs) at our institution. Two-hundred and two HCWs were evaluated, 158 subjects were screened for the nosocomial transmission of TB, and 143 subjects were eventually analyzed within a recently published contact investigation [7]. However, this study concluded that, fortunately, major nosocomial TB transmission from the source case did not occur. Nevertheless, the present case illustrates the increased risk of occupational TB transmission in health care [6,19].

Finally, the following basic implications for the general clinical practice emerge from this fatal case: a) whenever possible, aim at establishing a definite diagnosis before initiating treatment, particularly if cancer is suspected, and immediate or extensive therapeutic implications arise; b) easily treatable causes of disease should always be thoroughly considered and regularly re-evaluated, if applicable; c) copying and pasting medical diagnoses without critical reflection endangers patients and should therefore be strictly rejected.

## Conclusion

Awareness is the key point of diagnosing Pott's disease, an ancient but nowadays rare manifestation of extrapulmonary TB. Due to the devastating consequences of a missed diagnosis, MTB should be considered early in every case of spondylitis, intraspinal or paravertebral abscess. The presence of certain alarm signals like a prolonged history of progressive back pain, constitution symptoms or pulmonary nodules on a chest radiograph, particularly in the upper lobes, may guide the clinical suspicion. CT-guided percutaneous biopsy of the affected vertebral body and the consecutive microbiological and histological work-up including PCR may contribute to rapidly establishing the correct diagnosis.

## Consent

Written informed consent was obtained from the patient's widow for publication of this case report and any accompanying images. A copy of the written consent is available for review by the Editor-in-Chief of this journal.

## Competing interests

The authors declare that they have no competing interests.

## Authors' contributions

FCR took clinical care of the patient and drafted the manuscript. AT, AW and VN conducted and interpreted the pathological and radiological studies, respectively, and revised the manuscript critically for important intellectual content. HWD, GSW and GR took clinical care of the patient and revised the manuscript critically for important intellectual content. All authors read and approved the final manuscript.

## Supplementary Material

Additional file 1 **Table S1 - Summary of the patient's diagnostic and therapeutic in-hospital course**. This table summarizes the case patient's diagnostic and therapeutic course over more than three months at three different hospitals. Click here for file

## References

[B1] Brodhun B Altmann D Haas W [Report on the epidemiology of tuberculosis in Germany 2007] 2009 Berlin: Robert Koch-Institut (RKI)

[B2] Pott P The chirurgical works of Percivall Pott, F.R.S., surgeon to St. Bartholomew's Hospital, a new edition, with his last corrections. 1808 Clin Orthop Relat Res 2002 4 10 10.1097/00003086-200205000-00002 11964625

[B3] Nerlich AG Haas CJ Zink A Szeimies U Hagedorn HG Molecular evidence for tuberculosis in an ancient Egyptian mummy Lancet 1997 8 1404 10.1016/S0140-6736(05)65185-9 9365482

[B4] Turgut M Spinal tuberculosis (Pott's disease): its clinical presentation, surgical management, and outcome. A survey study on 694 patients Neurosurg Rev 2001 8 8 13 10.1007/PL00011973 11339471

[B5] Wang JT Hung CC Sheng WH Wang JY Chang SC Luh KT Prognosis of tuberculous meningitis in adults in the era of modern antituberculous chemotherapy J Microbiol Immunol Infect 2002 8 215 222 12542246

[B6] Diel R Seidler A Nienhaus A Rusch-Gerdes S Niemann S Occupational risk of tuberculosis transmission in a low incidence area Respir Res 2005 8 35 10.1186/1465-9921-6-35 15831092 PMC1087884

[B7] Ringshausen FC Schlosser S Nienhaus A Schablon A Schultze-Werninghaus G Rohde G In-hospital contact investigation among health care workers after exposure to smear-negative tuberculosis J Occup Med Toxicol 2009 8 11 10.1186/1745-6673-4-11 19505310 PMC2698921

[B8] Thwaites GE Nguyen DB Nguyen HD Hoang TQ Do TT Nguyen TC Nguyen QH Nguyen TT Nguyen NH Nguyen TN Nguyen NL Vu NT Cao HH Tran TH Pham PM Nguyen TD Stepniewska K White NJ Farrar JJ Dexamethasone for the treatment of tuberculous meningitis in adolescents and adults N Engl J Med 2004 8 1741 1751 10.1056/NEJMoa040573 15496623

[B9] Kotil K Alan MS Bilge T Medical management of Pott disease in the thoracic and lumbar spine: a prospective clinical study J Neurosurg Spine 2007 8 222 228 10.3171/spi.2007.6.3.222 17355021

[B10] Park DW Sohn JW Kim EH Cho DI Lee JH Kim KT Ha KY Jeon CH Shim DM Lee JS Lee JB Chun BC Kim MJ Outcome and management of spinal tuberculosis according to the severity of disease: a retrospective study of 137 adult patients at Korean teaching hospitals Spine 2007 8 E130 135 10.1097/01.brs.0000255216.54085.21 17304122

[B11] Nussbaum ES Rockswold GL Bergman TA Erickson DL Seljeskog EL Spinal tuberculosis: a diagnostic and management challenge J Neurosurg 1995 8 243 247 10.3171/jns.1995.83.2.0243 7616269

[B12] Le Page L Feydy A Rillardon L Dufour V Le Henanff A Tubach F Belmatoug N Zarrouk V Guigui P Fantin B Spinal tuberculosis: a longitudinal study with clinical, laboratory, and imaging outcomes Semin Arthritis Rheum 2006 8 124 129 10.1016/j.semarthrit.2006.04.007 16884974

[B13] Joseffer SS Cooper PR Modern imaging of spinal tuberculosis J Neurosurg Spine 2005 8 145 150 10.3171/spi.2005.2.2.0145 15739525

[B14] Pertuiset E Beaudreuil J Liote F Horusitzky A Kemiche F Richette P Clerc-Wyel D Cerf-Payrastre I Dorfmann H Glowinski J Crouzet J Bardin T Meyer O Dryll A Ziza JM Kahn MF Kuntz D Spinal tuberculosis in adults. A study of 103 cases in a developed country, 1980-1994 Medicine (Baltimore) 1999 8 309 320 10.1097/00005792-199909000-00003 10499072

[B15] Heyer CM Al-Hadari A Mueller KM Stachon A Nicolas V Effectiveness of CT-guided percutaneous biopsies of the spine: an analysis of 202 examinations Acad Radiol 2008 8 901 911 10.1016/j.acra.2008.01.020 18572127

[B16] Centers for Disease Control and Prevention Updated guidelines for the use of nucleic acid amplification tests in the diagnosis of tuberculosis MMWR Morb Mortal Wkly Rep 2009 8 7 10 19145221

[B17] Nene A Bhojraj S Results of nonsurgical treatment of thoracic spinal tuberculosis in adults Spine J 2005 8 79 84 10.1016/j.spinee.2004.05.255 15653088

[B18] Pai M Zwerling A Menzies D Systematic review: T-cell-based assays for the diagnosis of latent tuberculosis infection: an update Ann Intern Med 2008 8 177 184 18593687 10.7326/0003-4819-149-3-200808050-00241 PMC2951987

[B19] Menzies D Joshi R Pai M Risk of tuberculosis infection and disease associated with work in health care settings Int J Tuberc Lung Dis 2007 8 593 605 17519089

