# Supplementary material for: A fatal case of spinal tuberculosis mistaken for metastatic lung cancer: recalling ancient Pott's disease
Source: Ann Clin Microbiol Antimicrob. 2009 Nov 20;8:32. doi: 10.1186/1476-0711-8-32 (PMC2784744; doi:10.1186/1476-0711-8-32)
Supplement: Additional file 1 — Table S1 - Summary of the patient's diagnostic and therapeutic in-hospital course. This table summarizes the case patient's diagnostic and therapeutic course over more than three months at three different hospitals. [file 1476-0711-8-32-S1.PDF]

A fatal case of spinal tuberculosis mistaken for metastatic lung cancer: recalling ancient Pott's disease

Felix C. Ringshausen, Andrea Tannapfel, Volkmar Nicolas, Andreas Weber,

Hans-Werner Duchna, Gerhard Schultze-Werninghaus, Gernot Rohde

Additional file 1: Table S1 – Summary of the patient's diagnostic and therapeutic in-hospital course

Table S1 Summary of the patient's diagnostic and therapeutic in-hospital course

Departments	Dates	Procedures
**External Hospitals 1+2**	**Dec 8th 2006 – Jan 10th 2007**	
Emergency department (1)	Dec 8th 2006	Physical examination
Surgical ward (1)	Dec 8th-Dec 9th 2006	Nursing
Radiation oncology (2)	Dec 09th-Dec 22nd 2006, Jan 02nd-03rd, Jan 10th 2007	Radiation, repeated bronchoscopy and CT-guided transthoracic biopsy
Orthopedics (2)	Dec 18th 2006	Physical examination
**In-hospital Surgery/Neurotraumatology**	**Jan 10th – Feb 28th 2007**	
Emergency department	Jan 10th	Physical examination
Surgical ward	Jan 10th –Feb 28th	Nursing
Anesthesiology	Jan 12th, Feb 7th	Endotracheal anesthesia
OR and surgical ICU	Jan 12th, Feb 7th	Laminectomy, spondylodesis, surgical revision, critical care
Cardiology	Feb 17th	Transesophageal echocardiography
Urology	Feb 14th	Physical examination, ultrasound
**In-hospital Medicine/Pulmonary Care**	**Feb 28th – April 12th 2007**	
Medical ward	Feb 28th–Mar 8th, Mar 12th–Apr 12th	Nursing
Cardiology	Mar 1st	Transesophageal echocardiography
Gastroenterology	Mar 5th, Mar 7th	Ultrasound, percutaneous gastrostomy
Medical ICU	Mar 8th–Mar 12th	Airway management, critical care
Depts. with continuous contacts: Laboratory, Logopedics, Patient Services, Radiology, Rehabilitation Medicine		

The index case/patient was considered contagious at our institution from January 10th until March 7th 2007 (total 57 days), when isolation and antimycobacterial treatment was initiated. A consecutive large-scale in-hospital TB contact investigation among 143 health care workers is reported elsewhere [7]. ICU = intensive care unit; OR = operating room.
